# Allograft and Collagen Membrane Augmentation Procedures Preserve the Bone Level around Implants after Immediate Placement and Restoration

**DOI:** 10.3390/ijerph17041133

**Published:** 2020-02-11

**Authors:** Roni Kolerman, Nayrouz Qahaz, Eitan Barnea, Eitan Mijiritsky, Liat Chaushu, Haim Tal, Joseph Nissan

**Affiliations:** 1 Department of Periodontology and Dental Implantology, the Maurice and Gabriela Goldschleger School of Dental Medicine, Tel-Aviv University, Tel Aviv 39040, Israel; liat.natanel@gmail.com (L.C.); talhaim@post.tau.ac.il (H.T.); 2 Dentist, Kolerman Periodontal and Implant Clinic, Tel-Aviv 64389, Israel; nayrouz.qahaz@gmail.com; 3 Prosthodontist, Implant and Prosthodontic Clinic, Tel-Aviv 64163, Israel; drbarnea@gmail.com; 4 Department of Otolaryngology Head and Neck Surgery and Maxillofacial Surgery, Tel-Aviv Sourasky Medical Center, Sackler School of Medicine, Tel Aviv University, Tel Aviv 39040, Israel; mijiritsky@bezeqint.net; 5 Department of Oral Rehabilitation, the Maurice and Gabriela Goldschleger School of Dental Medicine, Tel-Aviv University, Tel Aviv 39040, Israel; nissandr@gmail.com

**Keywords:** immediate placement and restoration, radiographic bone loss, single-tooth implant

## Abstract

***Background***: Immediate implant placement and restoration (IPR), is a reliable treatment modality. ***Purpose***: This historical prospective study evaluated the medium-term outcomes of hard tissue after IPR in the anterior maxilla with simultaneous hard tissue augmentation. ***Methods***: Seventy-three patients treated with single-implant IPR in the anterior maxilla were followed for 1-8 years. Treatment involved, atraumatic extraction, immediate implant placement and abutment adaptation, followed by simultaneous augmentation with mineralized freeze-dried bone allograft (FDBA) particles to fill the gaps and restore the ridge. The surgical site was stabilized with a resorbable collagen membrane, followed by the connection of an acrylic provisional restoration. ***Results***: All implants osseointegrated during the follow-up period (mean, 34 ± 22 months). Radiographic evaluation of the distance between the implant shoulder (IS) and crestal bone level (CBL) was of 0.86 ± 0.86 mm and 0.8 ± 0.84 mm mesially and distally, respectively. Splitting the results into up to 3 years and 3–8 years of follow-up data, the corresponding values were 0.90 ± 0.83 and 0.68 ± 0.88 for the mesial aspect and 0.99 ± 0.87and 0.74 ± 0.83 for the distal aspect, respectively. Mean peri-implant probing depth was 3.63 mm (SD ± 1.06) and 16 implants (22%) presented at least one bleeding pocket of ≥5 mm (peri implant mucositis). Conclusions: The immediate replacement of a single maxillary tooth by implants combined with guided bone regeneration is a predictable treatment modality with favorable peri-implant bony response.

## 1. Introduction

Single-tooth immediate implant insertion and provisionalization, especially in the aesthetic zone, is a highly reliable treatment modality for replacing failing tooth [1]. Increasing patient expectations for reduced treatment time and improved esthetics and comfort have shifted research interest from implant survival toward optimal preservation of soft and hard tissue. Whenever possible, immediate placement and restoration (IPR) of implants is strongly recommended. There are a few factors that, in turn, may have an adverse effect on the final esthetic outcome. Recession of the marginal peri-implant mucosa [2,3] is one of the most significant of these factors. Being clearly related to the bone levels surrounding the implant [4]), maintenance of the soft tissue and underlying bone is of the utmost important [4].

Several factors have been claimed to influence the frequency and extent of marginal mucosal recession, including the peri-implant soft tissue biotype [3], connection of a provisional crown immediately following implant insertion [5], condition and thickness of the facial bone [6], orofacial position of the implant shoulder [7,8] and filling the gap and facial peri-implant marginal defects with autogenous bone or bone substitute grafts [9,10].

During implant placement into fresh extraction sockets, gaps usually remain between the implant surface and the inner wall of the facial plate of the bone. Moreover, following tooth extraction, the alveolar bone-supporting tooth undergoes constant significant atrophy during the first 3 months [11,12]. A marked reduction in the height of the alveolar ridge has been shown to consistently occur following tooth extraction; additionally, implant installation into the fresh extraction socket does not interfere with the process of bone modeling [13,14].

Different approaches have been advocated to preserve or improve the dimension and contour of the ridge following tooth extraction, including the use of various graft or filler materials, such as autografts, allografts, xenografts and synthetic grafts, and/or barrier membranes [15]. The rationale for the use of graft materials and membranes is to prevent the migration of cells from the gingival epithelium and connective tissues into this gap, thus permitting osteoprogenitor cells to occupy the established gap [16] and eventually regenerate the bone tissue, thus supporting osseointegration [17]. Moreover, these grafts are used to partially prevent horizontal and vertical resorption following extraction and to augment the buccal bone to achieve at least a 2-mm-thick bony plate buccal to the implant surface [18]. However, there is not enough evidence supporting or refuting the need for augmentation procedures concomitant with immediate implant placement [14] or whether any of the augmentation techniques are superior to the others [19,20,21].

Consequently, the purpose of this study was to validate the efficiency of an allogenic bone graft material and non-cross-linked collagen membrane in preventing marginal bone loss after the extraction of a single anterior maxillary tooth and treatment with IPR.

## 2. Materials and Methods

### 2.1. Patient Population

The present historical prospective single-arm study was designed to evaluate the medium-term changes in hard tissue around implants after immediate placement and restoration (IPR) in the anterior maxilla with simultaneous bone augmentation. A total of 90 consecutive patients have been referred to the senior author (R.K., periodontist) during the years 2010–2017 and treated with IPR with a single implant in the anterior maxilla (for an incisor, canine or premolar). Of these, 17 were not included in the final analysis. After verification of the implants osseointegration 6 months post-surgery, they continued with rehabilitation and maintenance/follow-up by the referring dentist. The remaining 73 patients, with available follow-up data were included in the study. All implants were restored according to the concept of immediate nonfunctional loading. The study was approved by the human ethics committee of Tel-Aviv University and patients signed an informed consent form. Manuscript preparation complied with the STROBE guidelines. Patients were considered for the study based on the following inclusion criteria:

1. Patients were at least 18 years old.
2. Extraction of a single tooth in the anterior maxilla was indicated; both adjacent teeth mesial and distal to the extraction site were present.
3. The alveolar process presented at least 5 mm of solid bone apical to the alveolus of the failing tooth to ensure initial implant stability.
4. After extraction, the extraction socket was preserved or was compromised (thinner than 1 mm, dehiscenced or fenestrated or combination of 2 of those defects), due to previous periodontal disease, peri-apical pathologies, fractured tooth or traumatic extraction.
5. The insertion torque of the implant reached at least 32 Ncm [21].

The exclusion criteria were as follows:

1. Heavy smokers (≥10) who had not committed to a smoking cessation protocol.
2. Poor plaque control or lack of oral hygiene compliance.
3. Untreated or uncontrolled periodontal disease [22].
4. Parafunctional habits, such as bruxism.
5. Uncontrolled diabetes.
6. Acute infection (with the present of pus or fistula) around the failing tooth.
7. Failure to achieve primary stability of at least 32 Ncm.

### 2.2. Surgical Protocol

**Figure 1:**
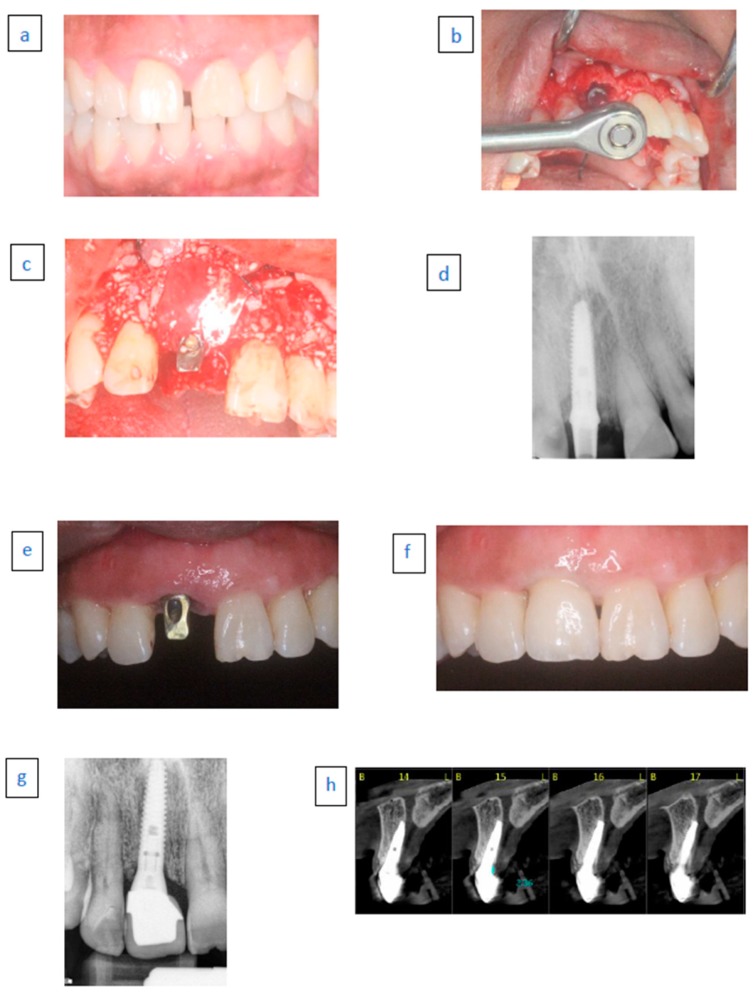
Clinical illustration of the implant placement and restoration (IPR) protocol. (**a**) Preoperative labial view of the failed maxillary right central incisor to be replaced with an implant. (**b**) Occlusal view after flap elevation, tooth extraction and implant placement in an appropriate three-dimensional position (**c**) Non-cross-linked native collagen membrane covering the freeze-dried bone allograft (FDBA) grafting material. (**d**) Periapical radiograph immediately after implant placement and simultaneous augmentation with mineralized FDBA particles and the resorbable membrane. (**e**) Buccal view of the final abutment 6 months after implant placement. (**f**) Buccal view of the definitive restoration 45 months after the final zirconia crown restoration. (**g**) Periapical radiograph after 45 months. (**h**) CT-scan at 45-months after crown installation demonstrating enlarged buccal bone and graft particles enclosed in the buccal free gingiva.

A thorough presurgical evaluation (Figure 1a) was performed and included a periodontal chart, smoking habits, periodontal diagnosis and full-mouth periapical radiographs. The following were evaluated preoperatively using periapical radiographs/computed tomograms: morphology of the alveolar process at the implant site, the root to be extracted, the location of the incisive foramen where relevant and the maxillary sinus and the presence of periapical pathologies. All patients underwent comprehensive periodontal cause-related therapy, including 1-6 sessions of oral hygiene instructions, scaling and root planning, whenever indicated and training, until a hygiene index (HI) [23] of less than 20% was achieved. The initial therapy was followed by surgical treatment if needed.

A one-minute rinse with chlorhexidine solution 0.2% (Tarodent mouthwash, Taro Pharmaceutical Industries, Ltd., Haifa, Israel) was used by the patients prior to surgery. Premedication with 875 mg of amoxicillin and clavulanic acid (Augmentin, Glaxo Smith Klein, Brentford, UK) was given one hour before surgery. Penicillin-sensitive patients were premedicated with clindamycin HCL (Dalacin-C, Pfizer NV/SA, Belgium) 150 mg bid starting one hour before surgery. Antibiotic administration (Augmentin) was continued for one week (Dalacin 150 mg × 4 per day was utilized in penicillin-sensitive patients) and analgesic administration (Naproxen sodium 275 mg, Narocin, Teva Pharm Ind., Ltd., Petah Tikva, Israel) was given for pain relief. The patients were instructed to rinse with 0.2% chlorhexidine twice a day for 3 weeks. All surgical procedures were performed by R.K. After the surgical site was anesthetized, the mucoperiosteal flaps were elevated, including intra crevicular incisions extending to the midfacial aspect of at least both adjacent teeth. This was followed by an atraumatic tooth extraction aiming to preserve the integrity of the extraction socket walls. Granulation tissue was removed using a spoon curette and a 3-mm diamond bur (Strauss Company, Raanana Israel). The drilling was conducted to the palatal wall. Osteotomy was intended to achieve as much implant engagement with the apical and palatal bone aspects of the extraction socket. Depending on the residual bone density, final drilling was performed using a drill measuring at least 1 mm in diameter less than the implant diameter. Final placement of the implant was achieved with an insertion torque of at least 32 Ncm using a torque-controlled ratchet (MIS Implants Technologies, Bar Lev industrial center, Israel). Screw-type bone-level titanium implants (Seven, Lance MIS Implants Technologies, Bar Lev industrial center, Israel) were used. Proper implant positioning was considered of pivotal importance, with the neighboring teeth essentially serving as a reference for optimal implant positioning (Figure 1b). A minimum distance of 1 mm (measured with a periodontal probe) in the mesiodistal direction between the implant shoulder and adjacent teeth was achieved in all cases. In the apicocoronal direction, the neck of the implant was flush with the palatal bone. In the orofacial dimension, an attempt was made to place the buccal neck of the implant at least 2 mm palatal to the buccal contour of neighboring teeth. An appropriate 0-25 degrees abutment with a gingival neck of 1–3 mm height was adapted, (not related to the socket configuration or defect morphology) followed by the application of 0.25–1 mm freeze-dried bone allograft (FDBA) particles (FDBA-Life-Net, Virginia, FL, USA) in the residual gap and in excess above the buccal wall. A resorbable collagen membrane (Bio-Gide, Geistlich Pharma AG, Wolhusen, Switzerland) was applied in a draping manner over the abutment and above the bone graft (Figure 1c). The buccal flap was coronally positioned after a periosteal releasing incision and sutured to the palatal flap using 4/0 sutures (Vicryl Rapid, Ethicon, Johnson Belgium).

### 2.3. Reconstructive Treatment Protocol

Abutment connection was verified radiographically (Figure 1d), followed by the adaptation of a prefabricated nonfunctional acrylic temporary crown (no occlusal contacts in Intercuspal Contact (IC) or protrusive or lateral movements). Sutures were removed after 7–10 days and were repeated when indicated.

Six months after implant placement, the temporary crown and abutments were removed, color-coded transfers (MIS Implants Technologies, Bar Lev, Israel) were adapted and radiographically verified. Impressions were taken using putty and wash silicone (Express, 3M ESPE Dental Products, St. Paul, MN, USA) employing the closed-tray technique and metal stock trays. A master model with a silicone image of the marginal gingiva was prepared and inter arch relations were recorded. At the following appointment, new abutments were connected (Figure 1e) and porcelain fused to metal or zirconia was tried (Figure 1f). Abutments were tightened to 25–35 Ncm (related to implant diameter) using a prosthetic ratchet. The permanent crown (Figure 1g) was cemented after occlusal adjustment and glazing with temporary cement (Temp-Bond, Kerr Corporation, 1717 West Collins Avenue, CA, USA). 

### 2.4. Clinical Follow-Up Examination

Patients were clinically followed for 6 and 12 months postoperatively and then annually thereafter. Patients participated in a personal maintenance program every 3-6 month performed by dental hygienists and included plaque index and probing depth and bleeding and bleeding index. measurement and recording. Bleeding index-consisted a dichotomous recording of the absence or presence of bleeding after probing of the gingival sulcus per implant (mesial, midfacial, distal and palatal). De-plaquing, scaling and root planning were performed as indicated.

### 2.5. Radiographic Evaluation

Postoperative periapical radiographs were obtained immediately after implant placement at the time of impression taking, at the final crown installation, at the annual follow-up examinations and once again at the time of final data collection during 2018 (cases 1–4). Standardized radiographs were obtained with the film kept parallel (Schick Technologies, Long Island, NY, USA) using plastic film holders while the X-ray beam was kept perpendicular.

### 2.6. Radiographic Data—Distance from the Implant Shoulder to the Coronal Bone-to-Implant Contact (DIB).

**Figure 2:**
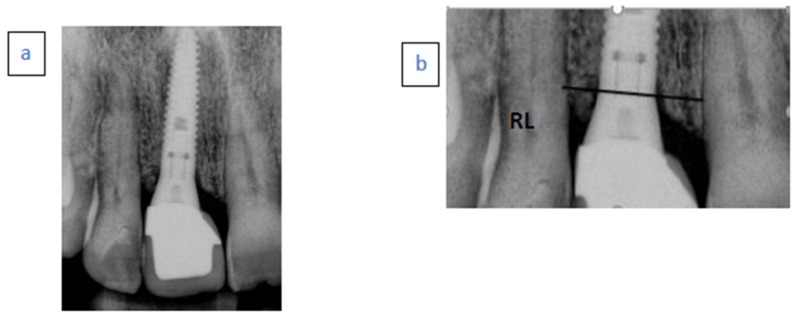
(**a**) Peri-apical radiograph 45 months after definitive crown placement. (**b**) Reference level (RL)was used to determine crestal bone level (CBL) values.

The distance from the mesial and distal alveolar bone crest to the implant shoulder that served as a reference level (RL), was digitally measured by computerized dental radiography based on parallel periapical X-rays (Schick Technologies, Long Island, NY) (Figure 2). The radiographic distortion was calculated by dividing the radiographic implant length by the actual implant length.

A positive value was given when the MBL was coronal to the RL. A negative value was given when the MBL was apical to the RL. The radiographic readings were performed by one experienced examiner (QN) not involved in the surgical or prosthetic treatment of the patient.

### 2.7. Implant Success

Peri-implant mucositis was defined as PD ≥5 mm with BOP and no bone loss.

Peri-implantitis was defined as implants showing more than 1.5 mm of bone loss during the first year and higher than additional 0.2 mm for each successive year [24].

Case 1

- A 65-year-old female patient.
- Nonsmoker with chronic advanced periodontitis.
- Case 1 (a) A hopeless second right premolar due to vertical root fracture.
- Case 1 (b) Immediate implant placement with temporary abutment.
- Case1 (c) Follow-up examination after 38 months.

Case 1

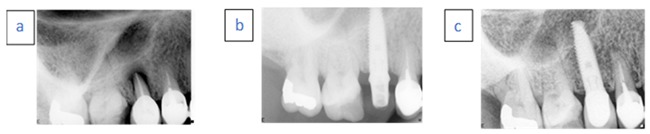



Case 2

- A 56-year-old male patient.
- Nonsmoker with chronic advanced periodontitis.
- Case 2 (a) A hopeless left maxillary first premolar tooth due to vertical fracture.
- Case 2 (b) Immediate implant placement with temporary abutment. Radiopaque material demonstrated above the implant neck.
- Case 2 (c) Follow-up examination after 36 months.

Case 2

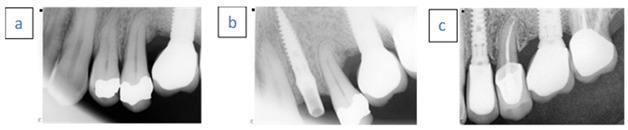



Case 3

- A 46-year-old female.
- Nonsmoker with generalized aggressive periodontitis.
- Case 3 (a) Hopeless left central incisor due to advanced bone loss.
- Case 3 (b) Immediate implant placement with temporary abutment. Radiopaque material demonstrated above the implant neck.
- Case 3 (c) Follow-up examination after 96 months.

Case 3

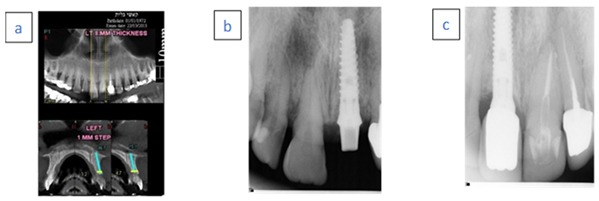



Case 4

- A 76-year-old female.
- Nonsmoker with chronic advanced periodontitis.
- Case 4 (a) Hopeless left lateral first incisor due to external root resorption.
- Case 4 (b) Immediate implant placement with temporary abutment.
- Case 4 (c) Follow-up examination after 24 months.

Case 4

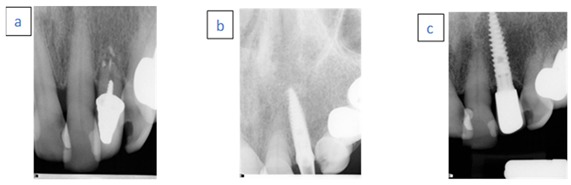



### 2.8. Statistical Analysis

Statistical analysis was performed with SPSS 20.0 statistical analysis software (SPSS, Inc., Chicago, IL, USA). Student’s t test and analysis of variance (ANOVA) were used to assess the differences in crestal bone level between groups of patients divided by age (≤/>56 years), sex, periodontal status, smoking habits, cause of tooth extraction and implant’s length/width. Due to the small sample size for mild chronic periodontitis and gingivitis, periodontal diagnoses were grouped together into the following two groups: a. gingivitis and mild to moderate chronic periodontitis; and b. advanced chronic and aggressive periodontitis. The Pearson correlation coefficient test was applied to test the correlation between age as a continuous variable and crestal bone level. *P* values < 0.05 were accepted as significant.

## 3. Results

The study population (Table 1 and Table 2) included 73 patients (33 males and 40 women) with ages ranging between 22 and 84 years (mean, 56.49 ± 14.56) who were treated according to a strict protocol consisting of single-tooth extraction, immediate implant placement, guided bone regeneration (GBR) and immediate restoration. Eleven implants replaced central incisors, 17 replaced lateral incisors, 6 replaced canines, 23 replaced first premolars and 16 implants were placed in the second premolar region. Forty-nine patients (67.1%) presented with chronic advanced adult periodontitis or aggressive periodontitis, whereas 24 (32.9%) were diagnosed with gingivitis or mild adult chronic periodontitis (Table 2). Twenty-nine (39.7%) were extracted due to periodontal disease, 19(26 %) due to root fracture, 22(30.1%) due to severe carious lesions and 3(4.1%) due to external root resorption (Table 2).

The relevant details of the study group, including sex, smoking status, implant site and dimensions (length and diameter) and abutment type, are presented in Table 1 and Table 2. The implant diameter varied between 3.3 and 5 mm (mean, 3.83 ± 0.43 mm) and the implant length varied between 10 and 16 mm (mean, 14.84 ± 1.66 mm). 

### 3.1. Radiographic Findings/DIB Values

**Figure 3:**
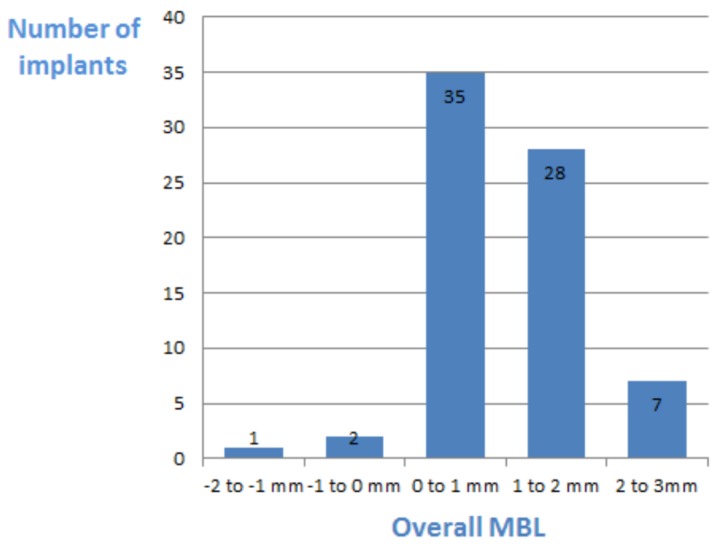
Frequency analysis of marginal bone level at the final follow-up examination.

At the time of final data collection, that is, after 12–96 months (mean, 34 ± 22), all 73 (100%) implants were deemed successful according to the Albrektsson criteria, showing no more than 1.5 mm of bone loss during the first year and up to an additional 0.2 mm for each successive year [24]. Moreover seventy (70) patients presented with marginal bone coronal (positive) or at the level of the implant shoulder (RL), whereas in three patients, bone level was apical to the implant shoulder (Figure 3).

Crestal bone level measurements revealed a mean mesial and distal bone level of 0.86 ± 0.86 mm (range, 0–3 mm) and 0.8 ± 0.84 mm (range, 0–3.3 mm), above the implant shoulder (RL) respectively. Splitting the study group into to the first 3 years and the following 3–8 years of follow up, the measurements yielded an average positive bone level of 0.90 ± 0.83 and 0.99 ± 0.87 mesial and distal to the implants and 0.68 ± 0.88 and 0.74 ± 0.83, respectively.

Paired t test and nonparametric signed t test for paired samples showed no difference between the CBL on the mesial or distal aspect of the implants. No difference was found in the CBL between light smokers and nonsmokers, whereas the bone level rate was significantly higher in males than in females (*p* = 0.04). CBL in advanced/aggressive periodontitis patients was higher than in the gingivitis/mild to moderate periodontitis: 1 ± 0.7 vs. 0.5 ± 0.9 respectively (*p* = 0.02) (Table 2). No correlation was found between the periodontal status, age or cause of extraction and the crestal bone level. There were no significant differences in the CBL between implants with different diameters or lengths.

### 3.2. Standard Soft Tissue Parameters

At the time of data collection, after 12–96 (mean, 34 ± 22) month observation period full mouth plaque index ranged between 7% to 50% with a mean of 18%.

Mean plaque score for implant supported crown was 18.17% (SD ± 0.69), mean PD at implant was 3.41 mm (SD ± 0.91) at the mesial aspect, 3.74 mm (SD ± 1.13) at the buccal, 3.46 mm (SD ± 1.35) at the distal and 3.90 mm (SD ± 1.23) at the palatal aspect, resulting in a mean peri-implant PD of 3.63 mm (SD ± 1.06). A total of 16 patients (22%) displayed mucositis (at least one bleeding pocket of ≥5 mm).

### 3.3. Technical Complications

During the follow-up period up to final crown installation a total of 25(34.2%) events of provisional crown loosening were observed in 20 patients. Twelve patients experienced 1 episode, five patients experienced 2 incidents and three patients experienced three incidents. Most provisional crowns could be re-cemented with temporary cement while five new crowns were made in 5 patients. Six abutment screws loosened before placement of the definitive crown. The provisional crown was removed, and the abutment was tightened without any further complications. Four definitive crown lost retention in 4 patients. All crowns were re-cemented by temporary cement. No ceramic fracture occurred.

### 3.4. Biological Complications

Sub nasal or suborbital hematoma during the first week after surgery occurred in 15 (20.5%) patients.

## 4. Discussion

After a mean follow-up period of 34 months, the cumulative implant success rate was 100%.

These results are in accordance with those of several studies demonstrating the high predictability of immediate implant placement and provisionalization with simultaneous bone augmentation [25,26,27]. In addition, a systematic review showed better crestal bone preservation around immediately (concomitant with extraction) placed implants than around implants placed in healed/native bone after 12 months of follow up [28].

Although most studies regarding IPR have shown bone loss of between −0.2 and −1.0 mm after the first year of function [28,29], controversially, other studies have reported minimal bone gain above the implant shoulder observed at the 1-year follow up [30,31]. Arora claimed a nonsignificant bone gain of 0.18 and 0.34 mm on mesial and distal implant aspects, respectively [31]. In their study, the gap between the implant and the inner extraction walls was filled with a slowly resorbing xenograft-increasing bone level [31]. 

In the current study all implants except three (70/73) presented bone gain indicating that the peri-implant marginal bone level can be well maintained or enhanced using the proposed treatment protocol. The results of the present study regarding positive crestal bone level are in accordance with a recent published meta-analysis comparing immediately placed implant combined with GBR vs. the use of bone grafts alone [32]. Despite presence of intact or dehisced sockets, the CBL was better preserved in IIP with bone graft and membrane compared with bone graft alone. The later finding is logical since membranes assist in complete graft containment without soft tissue downgrowth [32].

The low mean PD of implants (3.63 ± 1.06) at the last follow up examination in the present study indicated healthy peri-implant soft tissue and is in line with previous studies [25,26]. 

The maintenance of CBL in this study may be attributed to the surgical technique, which is characterized by the placement of mineralized FDBA granules into the gap and in excess over the buccal bone, followed by covering with a non–cross linked collagen membrane. Although this surgical technique has been used extensively in regenerative procedures [33,34], there is insufficient scientific evidence to support its efficacy in immediate implant placement [32,35].

A range of biomaterials, primarily bone xenografts and allografts, have been found to improve osseous volume [36]. Other preclinical [37] and clinical studies [38] have demonstrated that vertical bone loss is limited with the use of allografts covered with a resorbable collagen membrane. Moreover, aiming to compensate the volume reduction during the healing stage of the GBR procedure, over-augmentation was endorsed [21]. The low resorb ability of the graft can be advantageous, as it limits buccal bone resorption [39]. Collagen membranes were used to reduce the risk of infection if soft tissue dehiscence occurs postoperatively [40,41]. The osteoconductive properties of mineralized allografts have been previously described [42]. In comparison to autogenous bone and despite the lack of osteogenic properties of allografts [43], comparable volumetric results were achieved with or without autogenous bone when used to restore alveolar ridge deficiency [44].

The use of bone replacement grafts and temporary acrylic restorations has been shown to improve the soft tissue height and thickness compared to those in the control groups [45,46]. A possible explanation for these phenomena may be that the incorporation and encapsulation of graft particles in peri-implant soft tissue creates a “benign” foreign body reaction (Figure 1h), consequently improving the soft tissue dimensions [47].

Another possible explanation for our results is that the pressure of the lips and tongue on the provisional crown had generated stress at the implant shoulder, causing cell distortion directly within the peri-implant bone and initiating bone remodeling at the implant surface. This results in bone gain [48].

The findings of the present study confirm the positive contributions of the offered regenerative techniques in terms of osseous volume preservation during implant placement. Moreover, the fact that in the current study almost no bone loss occurred in the interproximal region and, on the contrary, positive bone crestal was commonly observed, may also indicate that a similar behavior occurs at the buccal and lingual aspects of the implant, as suggested by Botticelli et al. [49].

In the present study, we used screw- or conical-type, sand-blasted and acid-etched titanium implants placed at the bone level; these implants have several features that have been shown to minimize crestal bone loss, including crestal micro threads, a conical connection and a platform switch design. Although the rehabilitation protocol did not include the use of narrow abutments, the use of a slow resorbing osseoconductive FDBA material [50] may have contributed to the good results obtained regarding the CBL [51]. Clinical studies had demonstrated that submerged and trans-mucosal surgical technique with GBR yield similar good results with regard to degree of defect repair, implant survival and marginal bone levels, peri-implant soft tissue parameters and patient satisfaction [52].

Being historical prospective, this study presents two shortcomings—(a) the range of the follow-up period is relatively wide; and (b) the retrospective nature of data collection is associated with an inherent risk of missing data. Additionally, we did not include an aesthetic evaluation of the cases. However, due to the variations in the initial bone level of the hopeless teeth related to the different categories of periodontal status, cause for extraction and location in the maxillary arch, we considered aesthetic evaluations to be misleading. Another limitation is the lack of a control group; however, a recent publication posited that conducting randomized clinical trials to compare different timing protocols for implant placement after extraction would not be ethical [51]. The authors also noted that well-designed prospective case studies, in which defined inclusion and exclusion criteria are applied, as in the current study, represent most of the available evidence in the field of post extraction implant placement [53]. In favor of the study design - the same surgeon performed all procedures using the same implant system and grafting materials increased the homogeneity of our study group. Additionally, crestal bone level assessment as the primary outcome is directly related to the regenerative procedure performed.

More studies with homogeneity of the periodontal status and post-extraction defect characteristics are needed to confirm the efficacy of the GBR procedure over conventional implant placement or the use of bone grafts alone. The promising results regarding bone preservation and enhancement demonstrated in the current study may be better evaluated by using CT scans that will demonstrate more precisely the 3D morphology of the augmented area.

## 5. Conclusions

Within the limitations of this study, we demonstrate that the regenerative technique presented using mineralized FDBA particles combined with a non-cross-linked collagen membrane concomitant with immediate implant placement preserved the crestal bone level surrounding the implant.

## Figures and Tables

**Table 1:** Included patients and implants used.

Variable	NO	%
Gender		
Female	40	45.2
Male	33	54.8
smoker		
< 10 cigarettes per day	14	19.2
Nonsmokers	59	80.8
No. of implants	73	100
Implant length		
10 mm	1	1.3
11.5 mm	4	5.4
13 mm	20	27.4
16 mm	48	65.7
Implant platform		
3.3	19	26
3.75	26	35.61
4.2	25	34.24
5	3	4.1
Implant site, maxilla		
Central incisor	11	15
Lateral incisor	17	23.3
Canine	6	8.3
First premolar	23	31.5
Second premolar	16	21.9

**Table 2:** The association between patients’ and implants’ features and crestal bone level.

Variable	N (%)	Marginal Bone Level	*p* value
		Mean ± SD	
Gender			
Female	40 (54.8)	0.7 ± 0.9	0.04
Male	33 (45.2)	1.1 ± 0.7	
Age			
≤56 years	31 (42.5)	1 ± 0.8	NS
>56 years	42 (57.5)	0.8 ± 0.8	−0.2
Smoking			
No	59 (80.8)	0.8 ± 0.8	NS
Yes	14 (19.2)	1 ± 0.8	−0.5
Perio status			
1-gingivitis/mild to moderate periodontitis	24 (32.9)	0.5 ± 0.9	0.02
2-advanced/aggressive periodontitis	49 (67.1)	1 ± 0.7	
Reason for extraction			
Periodontitis	29 (39.7)	0.9 ± 0.8	
Caries	22 (30.1)	0.6 ± 0.7	NS
External resorption	3 (4.1)	1 ± 0.2	−0.3
Root fracture	19 (26)	1 ± 1	
Implant width			
3.3 mm	19 (26)	1 ± 0.8	
3.75 mm	26 (35.6)	0.8 ± 0.8	NS
4.2 mm	25 (34.2)	0.9 ± 0.8	−0.4
5 mm	3 (4.1)	0.1 ± 0.2	
Implant length			
10 mm	1 (1.4)	0	
11.5 mm	4 (5.4)	0.7 ± 0.8	NS
13 mm	20 (27.4)	0.9 ± 0.6	−0.7
16 mm	48 (65.7)	0.9 ± 0.9	
